# Digital Tooth Colour Acquisition: Agreement Between Spectrophotometric and Intraoral Scanner Measurements in Dental Students

**DOI:** 10.3390/dj14100636

**Published:** 2026-10-04

**Authors:** Alexia-Ecaterina Cârstea, Maria-Teodora Gheorghe, Yi Ze Chen, Nour-el hoda El-ghazoui, Lucian-Toma Ciocan, Mirela-Veronica Bucur, Alexandru-Titus Farcașiu, Marina Imre, Silviu-Mirel Pițuru, Ana Maria Cristina Țâncu

**Affiliations:** 1 Faculty of Dentistry, “Carol Davila” University of Medicine and Pharmacy, 37 Dionisie Lupu Street, District 2, 020021 Bucharest, Romania; alexia-ecaterina.carstea2023@stud.umfcd.ro (A.-E.C.); maria-teodora.gheorghe0721@stud.umfcd.ro (M.-T.G.); 2 Dentistry Department, Faculty of Medicine, San Pablo CEU University, 28668 Madrid, Spain; c.yize@usp.ceu.es; 3 Faculty of Dental Medicine, Mohammed V University in Rabat, Av. Mohamed El Jazouli, Madinat Al Irfane, Rabat BP 6212, Morocco; nourelhoda.elghazoui@um5r.ac.ma; 4 Discipline of Dental Prosthesis Technology, Faculty of Dentistry, “Carol Davila” University of Medicine and Pharmacy, 37 Dionisie Lupu Street, District 2, 020021 Bucharest, Romania; 5 Department of Teeth and Dental Arches Morphology and Dental Materials, Faculty of Dentistry, Carol Davila University of Medicine and Pharmacy, 17-21 Calea Plevnei Street, 010221 Bucharest, Romania; 6 Department of Prosthodontics, Faculty of Dentistry, “Carol Davila” University of Medicine and Pharmacy, 37 Dionisie Lupu Street, District 2, 020021 Bucharest, Romania; alexandru.farcasiu@umfcd.ro (A.-T.F.); marina.imre@umfcd.ro (M.I.); anamaria.tancu@umfcd.ro (A.M.C.Ț.); 7 Department of Professional Organization and Medical Legislation-Malpractice, Faculty of Dentistry, “Carol Davila” University of Medicine and Pharmacy, 37 Dionisie Lupu Street, District 2, 020021 Bucharest, Romania; silviu.pituru@umfcd.ro

**Keywords:** tooth colour, dental shade, spectrophotometry, intraoral scanner, digital dentistry, colour difference, inter-device agreement

## Abstract

**Objectives**. To describe the shade distribution and regional colour variation in a cohort of dental students and to quantify the agreement and shade-designation-derived colour difference between the VITA Easyshade V (VITA Zahnfabrik H. Rauter GmbH & Co. KG, Bad Saeckingen, Germany) spectrophotometer and the 3Shape TRIOS 5 (3Shape A/S, Copenhagen, Denmark) intraoral scanner at the cervical, middle and incisal thirds of the maxillary right central incisor, in both the VITA Classical and VITA 3D-Master systems. **Methods.** Tooth 11 was measured in 146 dental students with both devices at the three regions, the middle third serving as the representative body shade. The primary agreement outcome was the quadratic-weighted kappa of the Classical body shade, complemented by the paradox-robust Gwet AC1 and Brennan–Prediger coefficients; colour differences were obtained from published tab coordinates and not from coordinates measured on the teeth. **Results.** For the body shade (*n* = 144 teeth with an interpretable Classical designation from both instruments), exact agreement was 25.0% and Cohen’s kappa was near zero, the shades having clustered in a few light tabs. The primary outcome, the quadratic-weighted kappa, was 0.20 (95% confidence interval 0.09 to 0.34), with Gwet’s AC1 0.21 (0.12 to 0.27), Brennan–Prediger 0.19 (0.10 to 0.26) and an intraclass correlation coefficient, ICC(3,1), of 0.22 (0.03 to 0.39), none of them better than fair, and agreement on the 3D-Master lightness group was lower still. The scanner read lighter than the spectrophotometer by a mean of 1.80 value-ordered Classical units (Wilcoxon signed-rank *p* < 0.001) and by close to one lightness group on 3D-Master, and the two instruments distributed the shades differently at every region in both systems (Stuart–Maxwell *p* < 0.001). The scanner assigned a bleached 3D-Master shade in at least one region in 16.4% of participants against 1.4% for the spectrophotometer. On the Classical scale, agreement was weakest at the incisal third (Cohen’s kappa −0.03, quadratic-weighted kappa 0.03, Spearman 0.07, *p* = 0.42), where the mean signed difference was the smallest of any region, −0.39 units, and the dispersion the widest, with a mean absolute difference of 2.96 units and a standard deviation of 3.98 units. Colour varied across the three regions with both devices (Friedman *p* < 0.001), and the colour difference implied by the assigned tabs was above the 1.8-unit CIEDE2000 acceptability threshold for most tab pairs, carried by the lightness axis. **Conclusions.** The two instruments are not interchangeable. The scanner reads teeth as systematically lighter, most clearly at the cervical third and, on the Classical scale, least reliably at the incisal third, so a single device should be used throughout a case. With no independent reference standard, these data describe agreement and relative bias rather than accuracy. The scanner remains convenient for digital shade communication provided its lighter bias is taken into account.

## 1. Introduction

The colour of the natural dentition is one of the first features a patient notices and one of the hardest for a clinician to reproduce. Requests for tooth-coloured restorations and for whiter teeth have grown over the past two decades, and a restoration that is a good anatomical fit will still be considered a failure if its colour does not match the neighbouring teeth [1,2,3]. Accurate shade determination therefore sits at the centre of conservative, prosthetic and esthetic dentistry, and it is the step at which a great deal of clinical time is spent and which results in many laboratory remakes [4,5,6].

For most of the history of the profession, shade selection has been a visual task. The clinician holds a physical shade guide next to the tooth and chooses the tab that looks closest, usually working with the VITA Classical guide or the more systematically arranged VITA 3D-Master guide [7,8]. Visual matching is quick and needs no equipment, but it is also known to be inconsistent. The result depends on the light source, the surrounding colours, the experience of the observer, the angle of view, fatigue, and even the sex and age of the person making the choice, and agreement between two clinicians looking at the same tooth is often only moderate [9,10,11,12,13,14,15,16].

Instruments remove much of that subjectivity. Dental spectrophotometers measure the light reflected from the tooth across the visible spectrum and convert it into a shade designation or into CIE L*a*b* coordinates, and among them the VITA Easyshade family has become something close to a reference standard because of its repeatability and its agreement with visual assessment by experienced clinicians [17,18,19,20,21,22]. Spectrophotometers, colourimeters and digital cameras each have their own trade-offs, but the spectrophotometer is the device most studies use as the comparator when a new method is tested [23,24,25].

The newest of those methods arrives as a by-product of digital impressions. Intraoral scanners were designed to capture three-dimensional geometry, yet current models also record colour, and several manufacturers now offer an automated shade-measurement function that maps the scanned surface to VITA designations [26,27,28]. The appeal is obvious. A single pass of the scanner can capture the preparation, the arch and the shade at once, the data stay in the digital file, and the colour can be communicated to the laboratory without a separate instrument or a handwritten note [29,30,31,32]. The accuracy of these scanners for the geometric impression itself is now well documented, and interest is turning to whether the colour they capture is equally dependable, with machine-learning approaches already being explored to classify tooth shades automatically [33,34]. The 3Shape TRIOS family is one of the most widely used scanners with this capability, its shade function building on the same confocal optical principle used to reconstruct the surface, and the TRIOS 5 is its current wireless model [35,36,37,38,39].

Whether a shade read by a scanner can be trusted in the way a spectrophotometer reading is trusted remains an open question. The evidence gathered so far is mixed. Some reports describe reasonable agreement between scanners and spectrophotometers, while others find meaningful differences, and the results appear to depend on the scanner, the shade system, the region of the tooth and the population studied [40,41,42,43,44,45,46]. Very little of that work has examined the TRIOS 5 against the Easyshade V directly, across the cervical, middle and incisal thirds, and in both the VITA Classical and the 3D-Master systems at the same time.

The present study was designed to fill that gap in a real clinical cohort. Its aims were, first, to describe the distribution of natural tooth shades in a population of dental students, measured at three regions of the maxillary central incisor and summarised as an overall body shade; and second, to quantify the agreement between the VITA Easyshade V spectrophotometer and the 3Shape TRIOS 5 intraoral scanner for each region and for the body shade, in both shade systems, and to describe the regional colour gradient within the tooth. The working hypothesis was that the two instruments would agree closely enough to be used interchangeably and that neither would show a systematic tendency to read lighter or darker than the other.

## 2. Materials and Methods

### 2.1. Study Design

This was a cross-sectional, single-centre clinical study carried out at the Faculty of Dentistry of the Carol Davila University of Medicine and Pharmacy, Bucharest, Romania. The study was designed and reported in line with the STROBE recommendations for observational studies and, because its principal outcome is inter-device agreement, with the GRRAS recommendations for reliability and agreement studies [47,48].

### 2.2. Ethical Approval

The study protocol, the informed-consent form and the investigators’ documentation were reviewed and approved by the Research Ethics Subcommittee of the university (registration number 16374/12.06.2026). The study was conducted in accordance with the principles of the Declaration of Helsinki [49]. All participants received written and verbal information and provided written informed consent before any measurement.

### 2.3. Participants

Participants were dental students recruited through two parallel streams, a Romanian-language cohort and an English-language international cohort, which were pooled for the primary analysis. Eligible participants were at least 18 years old and had a sound, unrestored and non-carious maxillary right central incisor (tooth 11 in the FDI notation). Teeth carrying a restoration, a veneer or a fixed prosthesis on the labial surface, or showing caries, fluorosis or a visible enamel defect on the target tooth, were excluded, as were participants whose labial surface was obscured by a fixed orthodontic appliance. Any tooth-whitening procedure undertaken within the twelve months preceding the examination was a further exclusion criterion, because recent whitening alters the optical properties of enamel and would confound the bleached designations returned by the instruments. This criterion covered both routes of whitening, in-office professional bleaching and at-home or over-the-counter products, and eligibility in this respect was established by direct questioning at recruitment. Current smokers and users of other tobacco or nicotine products were also excluded [50], as were participants reporting habitual daily consumption of strongly staining beverages such as coffee, black tea or red wine, and participants with a known cause of intrinsic discolouration such as tetracycline staining, amelogenesis imperfecta or dentinogenesis imperfecta. A total of 146 participants, each contributing one tooth 11, were measured.

### 2.4. Device Specifications

The VITA Easyshade V (VITA Zahnfabrik H. Rauter GmbH & Co. KG, Bad Saeckingen, Germany) is a cordless, handheld dental spectrophotometer. Colour is recorded by a contact optical probe placed against the tooth surface, and a built-in spectrometer analyses the light reflected under illumination from an internal light-emitting-diode source. The instrument reports the VITA Classical A1 to D4 designation, the VITA SYSTEM 3D-MASTER designation including the bleached (0M) shades, and CIE L*a*b* coordinates, and it offers dedicated modes for a single tooth and for the separate regions of a tooth [17,51,52,53,54]. The instrument is thus capable of displaying CIE L*a*b* coordinates; those coordinates were not transcribed in the present study, for the reasons set out in Section 2.8.

The 3Shape TRIOS 5 (3Shape A/S, Copenhagen, Denmark), released in 2022, is a wireless, powder-free intraoral scanner that reconstructs a colour three-dimensional model of the dentition by confocal optical sectioning. In addition to geometry, the system provides an automated shade-measurement function that maps the captured colour of the labial surface to the VITA Classical and VITA 3D-Master systems for defined tooth regions [26,27,35].

### 2.5. Measurement Protocol

**Figure 1:**
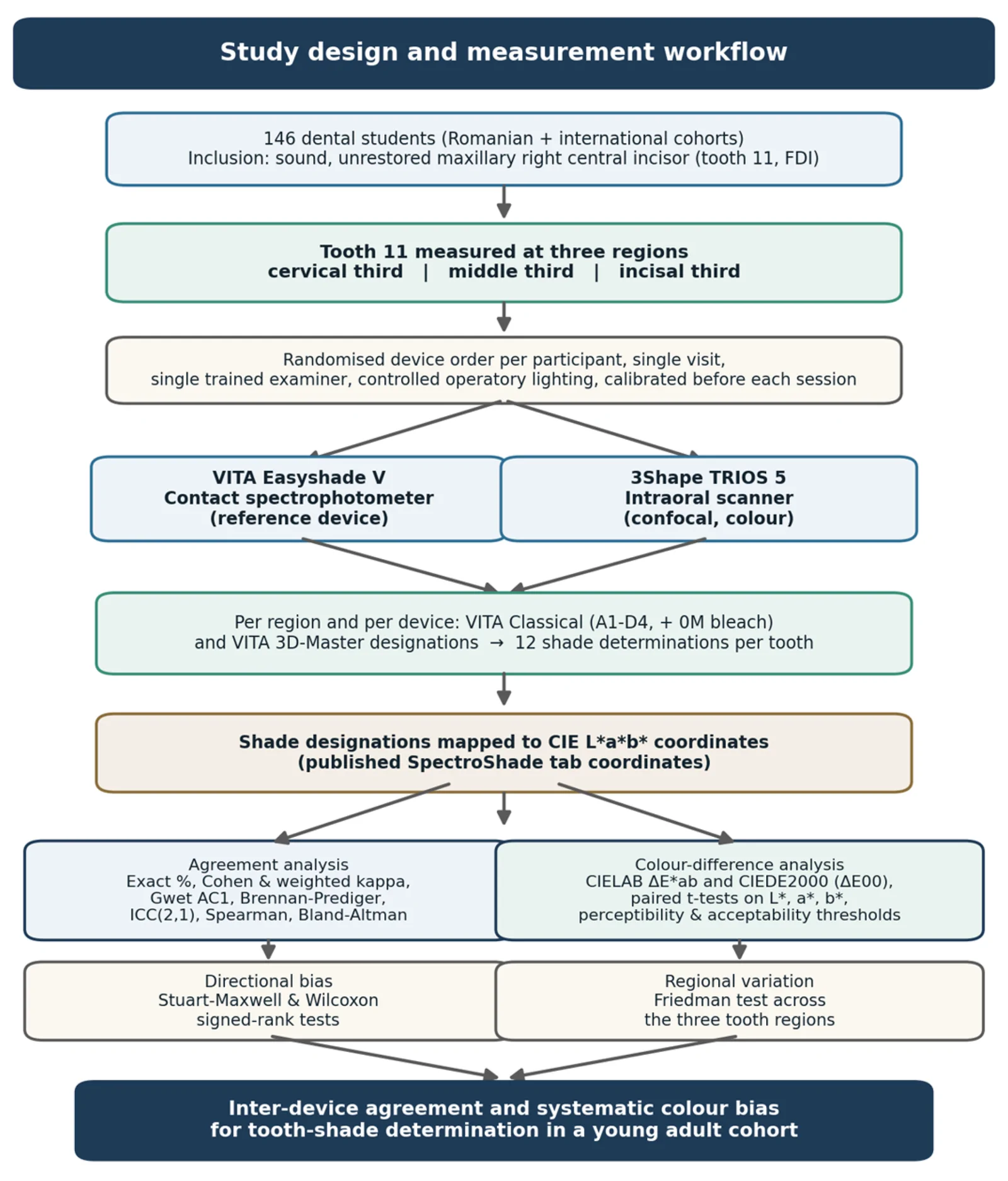
Overview of the study design and measurement workflow. Each maxillary right central incisor was measured by the VITA Easyshade V spectrophotometer and the 3Shape TRIOS 5 intraoral scanner, in randomised order, at three regions and in two shade systems; shade designations were then mapped to CIE L*a*b* coordinates for the colour-difference analysis.

Each tooth was measured by both devices, in a randomised order per participant, on the same visit, by a single trained examiner who also recorded the readings. The order of the two instruments was allocated from a computer-generated random sequence prepared before recruitment. That sequence was held separately from the case-report forms and carries no participant identifier, and the enrolment order was not retained in a form that can be matched to it, so the order actually applied to a given participant cannot be reconstructed from the surviving records. Teeth were cleaned before measurement; cleaning consisting of professional polishing of the labial surface with a rubber cup and a fluoride-free pumice-based prophylaxis paste at low speed, followed by rinsing with a water spray; the paste and the rubber cups were of a single brand and lot throughout the study so that surface preparation was identical for every participant, and no air-drying was applied at this stage, since polishing both lifts surface stain and dries the enamel and therefore had to be separated in time from the optical readings. Isolation was limited to a cheek retractor; neither cotton rolls nor rubber dam nor a continuous air stream was used, and the labial surface was not air-dried before either reading. After polishing, the tooth was left to rehydrate with the lips at rest for ten minutes before the first instrumental reading, and a fixed interval of five minutes separated the two readings, during which the retractor was removed and the lips were closed so that the surface could rewet [55]. Acquisition of the labial surface of tooth 11 with the intraoral scanner was limited to thirty seconds. The three measurement regions were standardised between the devices by dividing the height of the labial surface of the clinical crown into equal cervical, middle and incisal thirds. For the spectrophotometer, whose probe aperture is smaller than one-third of the crown height, the probe tip was centred mesiodistally within each third using the dedicated tooth-region mode of the instrument; for the intraoral scanner, the same three zones were identified on the reconstructed labial surface in the shade-measurement view before the designations were read. The two instruments were therefore referred to the same anatomical thirds, although the way in which the regions are fixed differs between them, as set out in Section 4.8. All determinations were taken under controlled operatory lighting, an environmental factor that is known to affect instrumental colour readings and was therefore held constant across participants [56,57]. Each device was calibrated according to the manufacturer instructions before every session. For every tooth, the VITA Classical and the VITA 3D-Master designations were recorded at the cervical third, the middle third and the incisal third with each instrument, giving twelve shade determinations per participant. The middle third served throughout as the representative body shade. No separate body-shade determination was made, so the body-shade and middle-third figures reported below are identical by definition and are presented once only. The overall study design and measurement workflow are summarised in Figure 1.

### 2.6. Examiner Calibration

All readings were taken by a single examiner, who was calibrated before the start of data collection. A subsample of 15 maxillary right central incisors was measured twice with each device, at two time points separated by at least 24 h, under the protocol described in Section 2.5. The exercise served procedural calibration only, and the duplicate readings were not carried into the analysis dataset, so no coefficient of intra-examiner reliability is reported; the consequence is stated in Section 4.8.

### 2.7. Coding and Statistical Analysis

Analyses were performed in Python 3.11 with the pandas, SciPy, statsmodels, pingouin and scikit-learn libraries. The Gwet’s AC1 and the Brennan–Prediger coefficients are not implemented in any of these libraries and were therefore coded directly in Python from the defining formulas of Gwet [58] and of Brennan and Prediger [59], and were verified against the worked examples published with those two sources. The code is available from the corresponding author on reasonable request. VITA Classical shades were placed on the value-ordered lightness scale (B1, A1, B2, D2, A2, C1, C2, D4, A3, D3, B3, A3.5, B4, C3, A4, C4), which arranges the tabs from lightest to darkest [8,60]. Because the intraoral scanner returned bleached designations (0M1 to 0M3) for teeth lighter than the lightest Classical tab, the value-ordered scale was extended at the light end so that 0M1, 0M2 and 0M3 preceded B1. 0M1, 0M2 and 0M3 are VITA 3D-Master bleached tabs and not VITA Classical tabs. Placing them in front of B1 extends the Classical value order by convention and not by any demonstrated colourimetric equivalence, and treating them as three equally spaced steps has no basis in colour science. Because the scanner returned a bleached designation for approximately one tooth in six, the influence of this convention on the Classical bias was examined under three alternative treatments of the bleached readings, reported in Section 3.3. VITA 3D-Master shades were summarised by their lightness group, from 0 (lightest) to 5 (darkest).

Inter-device agreement was assessed for each region and for the representative body shade, taken as the middle third, with the observed exact agreement, the Cohen’s kappa coefficient and the quadratic-weighted kappa coefficient interpreted against the Landis and Koch benchmarks, the percentage of pairs within one shade-guide unit, the Spearman rank correlation coefficient, and the two-way mixed-effects, absolute-agreement, single-measures intraclass correlation coefficient, denoted ICC(3,1) in the notation of Shrout and Fleiss and ICC(A,1) and in that of McGraw and Wong [61,62]. A mixed model is appropriate because the two instruments were purposefully selected rather than sampled at random from a population of devices, and absolute agreement was retained because systematic bias between them is the property at issue for interchangeability. The single-measures absolute-agreement coefficient has the same computational form under either rater model, so only the scope of the inference differs. The quadratic-weighted kappa and the ICC were computed on the value-ordered ranks, which were treated as an approximately interval lightness scale. Because natural tooth shades were expected to concentrate in a few light tabs, a distribution that is known to depress chance-corrected coefficients through the base-rate paradox, agreement was additionally quantified with the paradox-robust Gwet’s AC1 coefficient and the Brennan–Prediger coefficient [58,59,63]. The quadratic-weighted kappa for the body shade in the VITA Classical system was defined as the primary agreement outcome, with the Gwet’s AC1 and the Brennan–Prediger coefficients as its principal chance-corrected complements in view of the expected base-rate problem. All remaining coefficients and tests are complementary and characterise the pattern of disagreement rather than establish it. The designation of a primary outcome was made during revision of the manuscript and not before the analysis was carried out, and no adjustment was made for multiple comparisons; the complementary coefficients, the regional analyses and the sensitivity analyses are accordingly to be read as descriptive. The value-ordered rank difference between the two devices was displayed with a Bland–Altman plot, which shows the mean bias and the 95% limits of agreement between two measurement methods [64,65]. Because the value-ordered shade ranks form an ordinal scale on which consecutive categories do not represent equal colour intervals, this analysis is exploratory, and the limits of agreement are to be read as the spread of rank disagreement and not as a colourimetric tolerance [66]. Directional (systematic) disagreement was tested with the Wilcoxon signed-rank test on the value-ordered differences. The Stuart–Maxwell test was used separately to test marginal homogeneity, that is, whether the two instruments distribute the shades differently overall; it carries no information about the direction or the magnitude of any shift, which is read instead from the mean signed difference [67,68,69]. Bias and scatter were reported separately for each region, as the mean signed difference, the mean absolute difference and the standard deviation of the differences, because a small mean difference accompanied by a wide dispersion carries a different clinical meaning from a large and consistent offset. Proportional bias was tested formally by ordinary least-squares regression of the between-device difference on the mean of the two readings, the slope and its 95% confidence interval being reported for each region. Ninety-five per cent confidence intervals for the kappa coefficients and for the Gwet’s AC1 and Brennan–Prediger coefficients were obtained by bootstrap resampling with 2000 replicates. Regional colour variation within a device was tested with the Friedman test. A two-sided *p* value below 0.05 was considered significant. No a priori sample size calculation was performed. The cohort was a consecutively recruited convenience sample of dental students, no minimum size having been fixed in advance [70]. The precision actually achieved is therefore reported in place of an a priori target: on the 144 teeth contributing to it, the 95% confidence interval of the primary outcome, the quadratic-weighted kappa of the Classical body shade, had a width of 0.25 units, and that of the corresponding ICC(3,1) a width of 0.36 units [71].

### 2.8. Shade-Designation-Derived Colour-Difference Analysis

This analysis does not compare colours measured on the teeth. It compares the published coordinates of the tabs that the two instruments selected, so its output is a property of the pair of tab designations and not of the individual tooth: any two teeth to which the same pair of tabs was assigned necessarily receive exactly the same colour difference. To express the disagreement between the two devices in perceptual rather than purely ordinal terms, each recorded shade designation was mapped to its CIE L*a*b* coordinates using published mean coordinates of the VITA Classical and VITA 3D-Master tabs measured with a reference spectrophotometer, an approach that follows the standard colourimetric characterisation of shade guides [72,73,74]. For every tooth and region, the coordinates corresponding to the Easyshade and the TRIOS designations were compared, so that the analysis reflects the colour distance implied by the two devices selecting different tabs. The native chromatic coordinates were not exported because the study was designed around the shade designation: the designation is the quantity actually transmitted to the dental laboratory, and the shade-measurement function of the 3Shape TRIOS 5 returns a VITA tab only, with no route to a per-tooth L*a*b* export, so coordinates recorded from the spectrophotometer alone would not have produced a paired colourimetric dataset. The case-report form was accordingly built around the tab designations, and the coordinates displayed by the spectrophotometer were not transcribed at the chairside. Colour differences were computed with two formulas. The CIELAB difference is the Euclidean distance in the L^*^a^*^b^*^ space,

(1) $$\Delta E_{\mathrm{ab}}^{*}={\left[{\left(\Delta L^{*}\right)}^{2}+{\left(\Delta a^{*}\right)}^{2}+{\left(\Delta b^{*}\right)}^{2}\right]}^{1/2}$$

in which ΔL^*^, Δa^*^ and Δb^*^ are the differences between the coordinates of the two tabs [74]. The CIEDE2000 difference was computed in its lightness, chroma and hue form,

(2) $$\Delta E_{00}={\left\{{\left(\frac{\Delta L^{\prime }}{k_{L}S_{L}}\right)}^{2}+{\left(\frac{\Delta C^{\prime }}{k_{C}S_{C}}\right)}^{2}+{\left(\frac{\Delta H^{\prime }}{k_{H}S_{H}}\right)}^{2}+R_{T}\left(\frac{\Delta C^{\prime }}{k_{C}S_{C}}\right)\left(\frac{\Delta H^{\prime }}{k_{H}S_{H}}\right)\right\}}^{1/2}$$

in which Δ*L*′, Δ*C*′ and Δ*H*′ are the differences in lightness, in the corrected chroma and in the corrected hue, *S_L_*, *S_C_* and *S_H_* are the weighting functions for those three attributes, *R_T_* is the rotation term correcting the interaction between chroma and hue differences in the blue region, and the parametric factors are set to *k_L_* = *k_C_* = *k_H_* = 1. Chroma and hue were obtained from the Cartesian coordinates as

(3) $$C_{\mathrm{ab}}^{*}={\left[{\left(a^{*}\right)}^{2}+{\left(b^{*}\right)}^{2}\right]}^{1/2},\;\;h_{\mathrm{ab}}=\arctan\left(\frac{b^{*}}{a^{*}}\right)$$

before the CIEDE2000 corrections were applied [60,74]. The formula is that of Luo, Cui and Rigg and the implementation followed the notes of Sharma, Wu and Dalal, including their treatment of the hue-angle discontinuity [75,76].

The resulting colour differences were interpreted against the perceptibility and acceptability thresholds established for dentistry, taken as 1.2 and 2.7 units for the CIELAB difference and 0.8 and 1.8 units for the CIEDE2000 difference [77,78,79,80]. Those thresholds were established for directly determined colour differences. Because the present values are derived from the coordinates of assigned shade tabs, every comparison with them is an estimate conditional on the tab-mapping procedure rather than a measured proportion of clinically unacceptable teeth. No significance test is applied to the mean L*, a* and b* differences between the two devices. Each coordinate is a deterministic function of the assigned tab, so such a test would re-express the marginal shift already established by the Stuart–Maxwell test and would provide no independent information. Readings that could not be mapped were excluded from the colour-difference computation only. Two situations arose: scanner VITA Classical bleached designations, for which the Classical coordinate table contains no entry, and a small number of designations lying outside the published coordinate tables. The number of teeth excluded for each reason, by region and shade system, is given in Table 1, and the numbers analysed at each region are reported with the results. The colour differences are reported in full in Supplementary Material S1 (Table S1 and Figure S1).

## 3. Results

### 3.1. Study Population

The 146 participants had a mean age of 20.3 years (standard deviation 1.3, range 18 to 23). Of the 142 participants with complete demographic data, 89 (62.7%) were women and 53 (37.3%) were men, and 120 (84.5%) were of urban residence; of all 146 participants, 113 (77.4%) belonged to the Romanian cohort and 33 (22.6%) to the international cohort. The characteristics of the sample are summarised in Table 2.

### 3.2. Distribution of Tooth Shades

**Figure 2:**
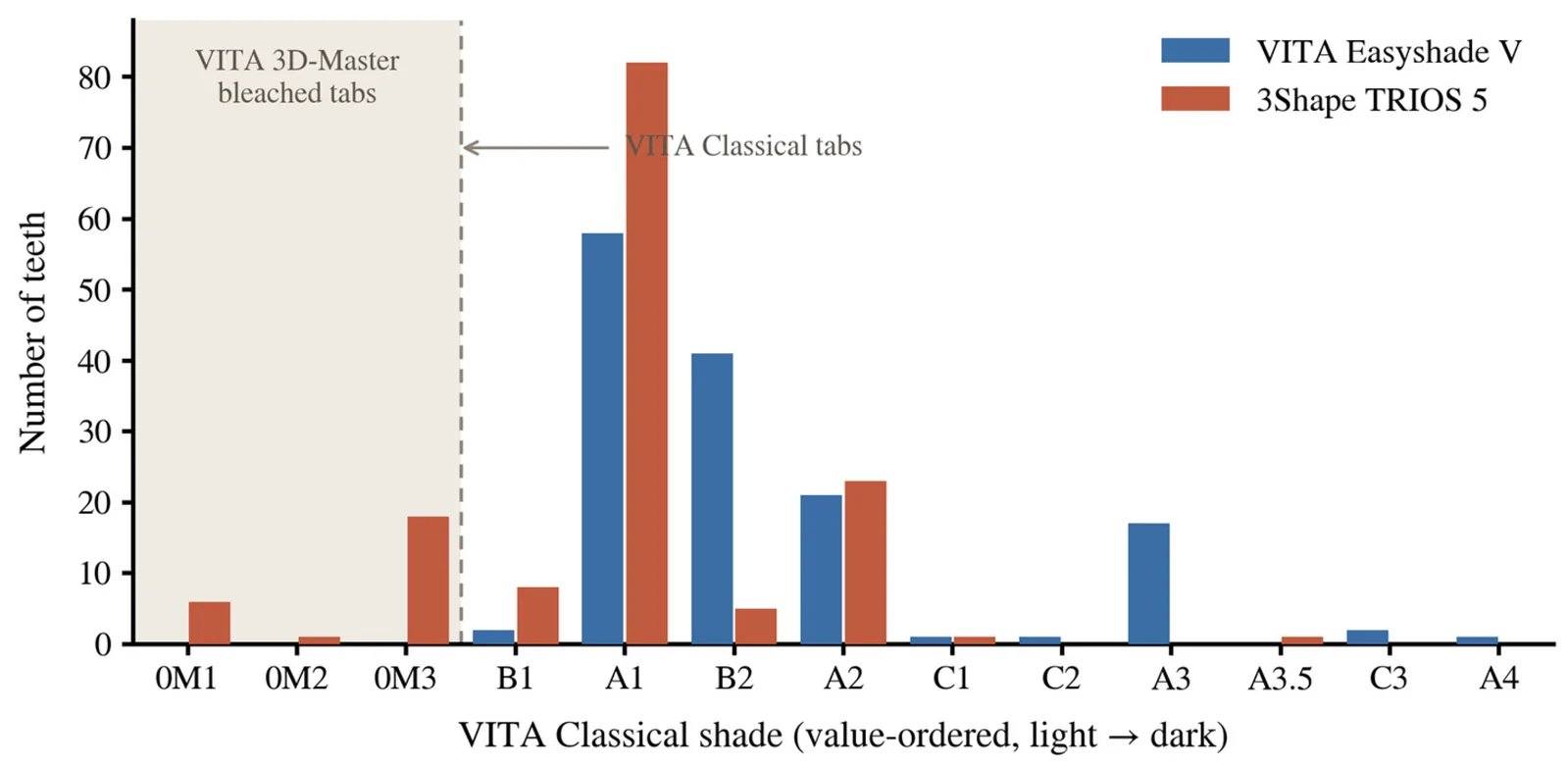
Distribution of body (middle-third) VITA Classical shades recorded by the two devices. Shades are arranged from lightest to darkest on the value-ordered scale defined in Section 2.7; the bleached shades 0M1 to 0M3 lie to the left of B1. These three tabs belong to the VITA 3D-Master system and are shown on a shaded ground, separated from the VITA Classical tabs by a broken rule, because their placement to the left of B1 is a convention adopted for the value ordering and is not a colourimetric equivalence, as explained in Section 2.7. The bars are counts of teeth returning each designation at the middle third; they sum to 144 for the spectrophotometer and 145 for the scanner, the difference being the single tooth for which no interpretable spectrophotometer designation was returned at that region.

With the spectrophotometer, the most frequent body (middle-third) Classical shade was A1 (40.0%), followed by B2 (28.3%), A2 (14.5%) and A3 (11.7%). With the intraoral scanner, the body Classical distribution was shifted toward lighter tabs, with A1 in 56.6%, A2 in 15.9%, the bleached shade 0M3 in 12.4%, B1 in 5.5% and 0M1 in 4.1% (Figure 2). In the 3D-Master system, the most frequent body shade with the spectrophotometer was 2M2 (30.1%), followed by 2L1.5 (16.4%) and 2M1 (10.3%), whereas with the scanner, it was 1M2 (50.0%), followed by 1M1 (26.7%) and 0M3 (12.3%). The scanner assigned a bleached 3D-Master shade in at least one region in 24 participants (16.4%), against 2 participants (1.4%) for the spectrophotometer. The pooled mean lightness group values were 2.22 for the spectrophotometer and 1.31 for the scanner, and the pooled mean Classical value ranks were 7.64 and 6.21, respectively. The bleached proportions that can be read off the two lists above, 16.5% on the Classical scale (0M3 in 12.4% and 0M1 in 4.1%) and 12.3% for 0M3 on the 3D-Master scale, refer to the same set of scanner readings but are not directly comparable as printed, for three reasons. (1) The denominators differ, there being 145 interpretable scanner readings on the Classical scale at the middle third and 146 on the 3D-Master scale, while the spectrophotometer returned 145 interpretable Classical readings at that region. (2) Each list reports only the most frequent designations, so the less-frequent bleached tabs, 0M2 on the Classical scale and 0M1 on the 3D-Master scale, are absent from the totals that can be formed from them. (3) One participant received a bleached Classical designation together with a non-bleached 3D-Master designation from the scanner at the same region; that record was checked against the source documentation and retained as recorded. The complete count of scanner bleached readings that could not be mapped to VITA Classical coordinates is given by region in Table 1. Three denominators therefore apply to the middle third on the Classical scale and should not be confused with one another. The Classical distributions given above are computed, for each instrument, on the 145 teeth for which that instrument returned an interpretable Classical designation, the two sets of 145 not being identical because the missing reading is a different tooth for each device. The agreement statistics of Table 3 and the sensitivity analysis of Section 3.3 are computed on the 144 teeth for which both instruments returned one at that region. The colour-difference computation of Section 3.5 is confined to the 119 teeth whose designations could both be mapped to published tab coordinates, the exclusions being set out in Table 1.

### 3.3. Inter-Device Agreement

Agreement between the two devices was fair at best and was accompanied by a strong directional bias (Table 3). For the Classical body shade, exact agreement was 25.0% and Cohen’s kappa was −0.01 (95% confidence interval −0.07 to 0.06); the near-zero Cohen’s kappa reflected the concentration of shades in a few light tabs, and the paradox-robust coefficients placed agreement at the boundary of the slight and fair ranges (Gwet’s AC1 0.21, 95% confidence interval 0.12 to 0.27; Brennan–Prediger 0.19, 0.10 to 0.26), consistent with the quadratic-weighted kappa of 0.20 (0.09 to 0.34) and ICC(3,1) of 0.22 (0.03 to 0.39). The proportion of pairs within one shade-guide unit was 52.1%, and the Spearman coefficient was 0.36 (*p* < 0.001). The mean signed difference was −1.80 value-ordered units; the Wilcoxon signed-rank test indicated a systematic difference in that direction, and the Stuart–Maxwell test a departure from marginal homogeneity (*p* < 0.001 for both). Agreement was highest at the cervical third (exact 33.1%, Cohen’s kappa 0.15, weighted kappa 0.28, Gwet’s AC1 0.29, 95% confidence interval 0.21 to 0.37, mean difference −2.16) and lowest at the incisal third (exact 8.3%, Cohen’s kappa −0.03, weighted kappa 0.03, Gwet’s AC1 0.03, −0.02 to 0.08, Spearman coefficient 0.07, *p* = 0.42).

Bias and scatter behaved differently along the tooth. The mean signed difference is reported for every region and both shade systems, together with the mean absolute difference and the standard deviation of the differences on the Classical scale, in Table 3. On the Classical scale the incisal third combines the smallest systematic offset of any region with the widest dispersion, which is qualitatively different from the consistent lightening observed cervically. It is scatter rather than bias, and it cannot be removed by applying a fixed correction.

**Table 3:** Inter-device agreement between the VITA Easyshade V and the 3Shape TRIOS 5 for tooth 11, by region and shade system. The Gwet’s AC1 and Brennan–Prediger coefficients are chance-corrected agreement coefficients robust to the base-rate paradox produced by the concentration of shades in a few light tabs; both are reported with bootstrap 95% confidence intervals (2000 replicates). The body shade is, by definition, the middle third and is therefore reported once, in the middle-third row of each block. Mean diff is the mean signed difference (TRIOS minus Easyshade) in scale units, with negative values indicating lighter scanner readings. Mean abs diff and SD of diff are the mean absolute difference and the standard deviation of those differences, given for the VITA Classical scale only; they were not computed on the 3D-Master lightness group. n is the number of teeth for which both instruments returned an interpretable designation in that shade system at that region, and is the denominator of every statistic in the row. The Stuart–Maxwell test, described in Section 2.7, tests marginal homogeneity alone and is not a test of bias: at the incisal third it is significant alongside a mean difference of only −0.39 units, because what separates the instruments there is the dispersion rather than a systematic offset.

Region	n	Exact (%)	Cohen’s κ (95% CI)	Weighted κ	Gwet’s AC1/Brennan–Prediger (95% CI)	Spearman ρ	ICC(3,1) (95% CI)	Mean Diff	Mean Abs Diff	SD of Diff	Stuart–Maxwell *p*
***VITA Classical (value-ordered lightness units; 19-step extended scale)***											
Cervical	145	33.1	0.15 (0.07 to 0.23)	0.28	0.29 (0.21 to 0.37)/0.28 (0.19 to 0.36)	0.35	0.28 (0.08 to 0.46)	−2.16	2.93	3.63	<0.001
Middle third (=body shade)	144	25.0	−0.01 (−0.07 to 0.06)	0.20	0.21 (0.12 to 0.27)/0.19 (0.10 to 0.26)	0.36	0.22 (0.03 to 0.39)	−1.80	2.24	2.86	<0.001
Incisal	145	8.3	−0.03 (−0.06 to 0.01)	0.03	0.03 (−0.02 to 0.08)/0.02 (−0.03 to 0.07)	0.07	0.04 (−0.13 to 0.20)	−0.39	2.96	3.98	<0.001
***VITA 3D-Master (exact agreement on the full shade designation; all other statistics on the lightness group)***											
Cervical	145	15.2	0.01 (−0.03 to 0.06)	0.10	0.14 (0.04 to 0.21)/0.09 (−0.02 to 0.16)	0.09	0.10 (−0.05 to 0.26)	−0.87			<0.001
Middle third (=body shade)	146	9.6	0.03 (−0.01 to 0.07)	0.05	0.04 (−0.05 to 0.12)/−0.02 (−0.12 to 0.06)	0.17	0.05 (−0.05 to 0.16)	−0.97			<0.001
Incisal	146	11.6	0.02 (−0.02 to 0.07)	0.15	0.11 (0.03 to 0.20)/0.09 (0.01 to 0.17)	0.20	0.15 (−0.01 to 0.31)	−0.91			<0.001

**Figure 3:**
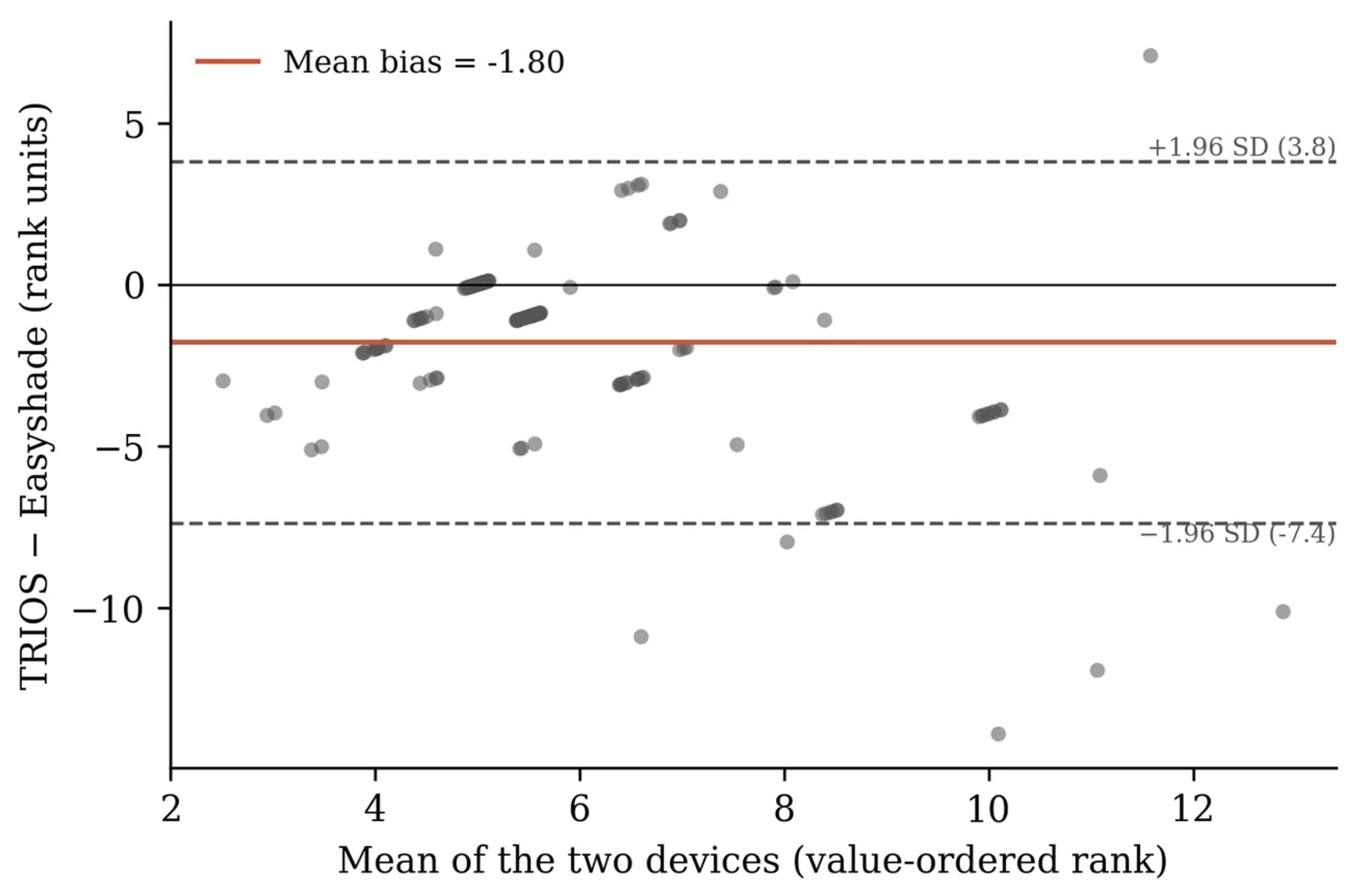
Bland–Altman plot of the body (middle-third) VITA Classical value rank. The solid line is the mean bias (TRIOS minus Easyshade), and the dashed lines are the 95% limits of agreement. Points are jittered to reduce overlap. The analysis is exploratory: the value-ordered ranks form an ordinal scale whose consecutive steps are not equally spaced in colourimetric terms, so the limits of agreement describe the spread of rank disagreement and not a colour tolerance.

The impression conveyed by Figure 3, that the disagreement widens for the darker teeth, was tested formally by regressing the between-device difference on the mean of the two readings. The slope was −0.580 (95% confidence interval −0.809 to −0.351, *p* < 0.001) at the body region, −0.491 (−0.706 to −0.276) at the cervical third and −0.511 (−0.818 to −0.204) at the incisal third, so a proportional component of the bias is confirmed. This result carries an important qualification: the scanner readings are compressed against the light end of a bounded ordinal scale, and a negative slope of this kind is, in part, the expected consequence of regression to the mean, so the magnitude of the proportional effect is likely to be overstated by the regression.

Because the placement of the bleached tabs in front of B1 is conventional, the Classical bias at the body region was recomputed under three treatments of those readings. The analysis is confined to the 144 teeth for which both instruments returned an interpretable Classical designation at that region. With 0M1 to 0M3 treated as three equal steps, the primary convention, the mean signed difference, was −1.80 units (n = 144); with the three bleached tabs collapsed into a single step immediately lighter than B1, it was −1.71 units (n = 144); and with all bleached readings excluded, it was −1.37 units (n = 119). A fourth variant, in which the three bleached tabs are spaced according to their published L* values, was not adopted, because the coordinate source used here does not report the 0M tabs and the VITA Classical tabs on a common measurement basis, so the resulting estimate would carry the properties of that source into the primary result. Across the three conventions reported, the treatment of the bleached readings accounts for at most about a quarter of the observed Classical bias, and neither the direction nor the statistical significance of the difference depends on the choice; the excluded-readings variant additionally rests on a reduced sample from which the most divergent teeth are absent.

The order in which the two instruments were applied was randomised for each participant, but the realised sequence was not recorded in the analysis dataset, so neither the order-stratified analysis nor the device-by-order interaction test could be performed; this is treated as an open limitation in Section 4.4 and Section 4.8.

**Figure 4:**
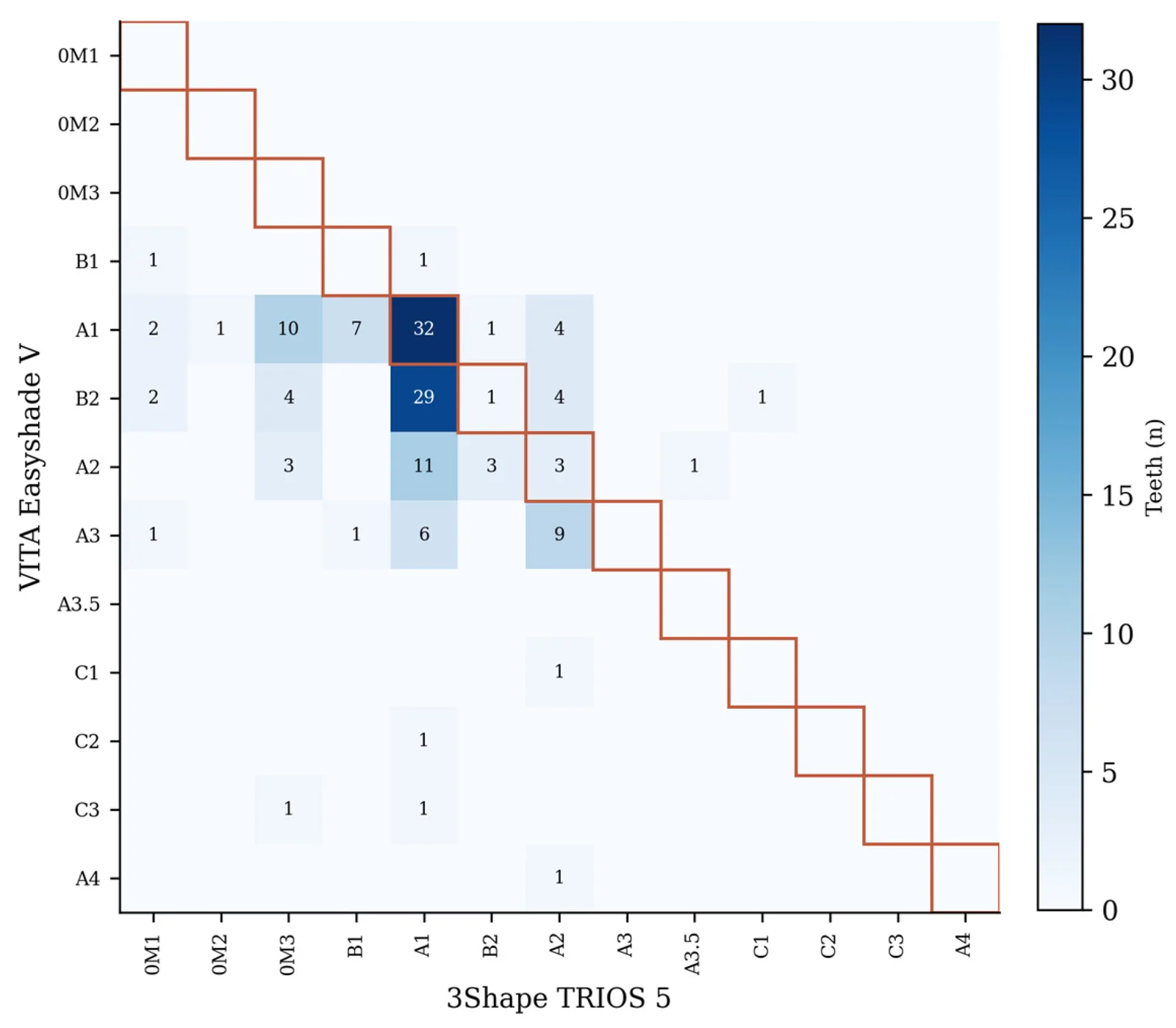
Cross-tabulation of body (middle-third) VITA Classical shades. Rows are the spectrophotometer and columns the intraoral scanner; the outlined cells are the diagonal of exact agreement. Both axes run from lightest to darkest, so cells below and to the left of the diagonal correspond to lighter scanner readings.

For the 3D-Master system, exact agreement of the full shade was 9.6% to 15.2%, and Cohen’s kappa was 0.01 to 0.03 across regions (the exact-agreement column of the 3D-Master block of Table 3 refers to the complete shade designation, whereas every other statistic in that block is computed on the lightness group). On the lightness group, the mean signed difference between the scanner and the spectrophotometer was −0.87 to −0.97 groups, with a significant departure from marginal homogeneity at every region (Stuart–Maxwell *p* < 0.001). The scanner recorded a lighter shade than the spectrophotometer in 53.8% of teeth at the cervical third, 66.7% at the middle third and 50.3% at the incisal third on the Classical scale, and in 72.4%, 80.1% and 61.6%, respectively, on the 3D-Master lightness group. The Bland–Altman analysis of the body value rank (Figure 3), presented here as an exploratory description of the disagreement, showed a mean bias of −1.80 units with wide limits of agreement (−7.4 to +3.8), and the cross-tabulation of body Classical shades (Figure 4) confirmed that most disagreements lay on the lighter side of the diagonal for the scanner. The extended Classical scale used here contains 19 value-ordered steps, so limits of agreement of −7.4 to +3.8 span 11.2 steps, approximately 59% of the entire scale. An interval of that width is the clearest single expression of how far the two instruments may diverge on an individual tooth.

### 3.4. Regional Colour Variation

**Figure 5:**
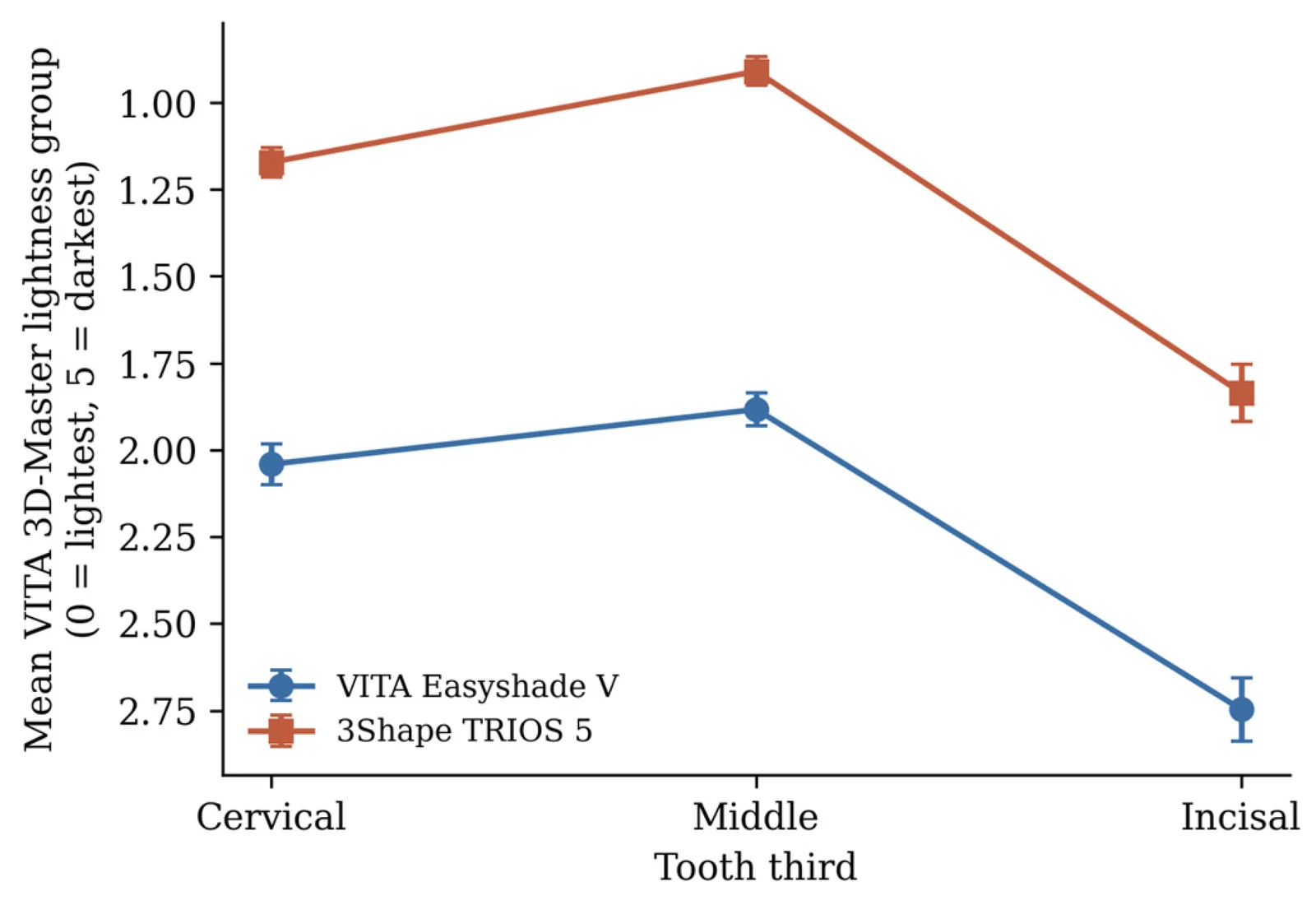
Mean VITA 3D-Master lightness group by tooth region and device. Lower values are lighter. Error bars are the standard error of the mean; the vertical axis is inverted so that lighter readings appear higher.

Colour varied across the three regions for both devices (Friedman test, *p* < 0.001 in every case). On the Classical value rank, the cervical third had the highest (darkest) mean rank (spectrophotometer 9.61 at the cervical third, 6.90 at the middle third and 6.44 at the incisal third; scanner 7.46, 5.12 and 6.07 respectively). On the 3D-Master lightness group, the incisal third had the highest mean value (spectrophotometer 2.04, 1.88 and 2.75; scanner 1.17, 0.91 and 1.84) (Figure 5). These means are expressed on the zero-based lightness-group coding defined in Section 2.7. So expressed, they average 2.22 for the spectrophotometer and 1.31 for the scanner, which reproduces the pooled values given in Section 3.2 exactly, and their maximum of 2.75 lies within the vertical axis of Figure 5.

### 3.5. Colour Differences Implied by the Shade Designations

The values in this subsection are not colour differences measured on the teeth. They are the colour distances between the published coordinates of the tabs that the two instruments assigned, so they restate the disagreement of Table 3 in colourimetric units, and the standard deviations reported below describe how often the assigned tab pairs differed and not how much the colour of the teeth varied. The proportions described as exceeding the acceptability threshold are accordingly estimates conditional on the shade-designation mapping. Expressed in these terms, the difference between the two devices was large and consistent (Table S1). In the VITA 3D-Master system, the mean CIEDE2000 colour difference between the shades chosen by the two instruments was 3.83 at the cervical third, 4.15 at the middle third and 4.82 at the incisal third, and the corresponding CIELAB differences were 5.59, 5.93 and 6.55; the colour difference exceeded the 1.8-unit acceptability threshold for CIEDE2000 in 81.8%, 87.6% and 86.3% of the assigned tab pairs, respectively (Figure S1). In the VITA Classical system, the mean CIEDE2000 differences were smaller but still above the acceptability threshold, at 2.32, 2.15 and 3.52 across the three regions, exceeding that threshold in 61.0%, 57.1% and 65.7% of the assigned tab pairs. These percentages count pairs of assigned tabs whose published coordinates differ by more than the threshold, and not teeth judged unacceptable by any observer. The number of teeth contributing to these figures differs between the two shade systems, and the exclusions are set out in Table 1. In the VITA Classical system, the excluded teeth are almost entirely those for which the scanner returned a bleached designation, which the Classical coordinate table cannot represent: 4 of 5 exclusions at the cervical third, 25 of 27 at the middle third and 11 of 12 at the incisal third. Those are precisely the teeth on which the two instruments diverged most, so at the cervical and middle thirds, the Classical colour differences reported here are conservative and understate the separation between the devices, most markedly at the middle third. At the incisal third, the exclusion does not simply attenuate the difference: the mean L* of the remaining tabs is negative; that is, the tabs the scanner assigned are the darker ones, which is the reverse of the direction seen on the full sample in Table 3. In neither case should the smaller Classical values be read as evidence of closer agreement in that system.

The lightness axis accounts for most of the separation. On the 3D-Master coordinates, the tabs chosen by the scanner were lighter than those chosen by the spectrophotometer by a mean L* of 3.65 at the cervical third, 4.37 at the middle third and 3.86 at the incisal third, and were slightly less red and less yellow, the mean a* differences lying within about one unit and the mean b* differences between −2.07 and −2.92. On the Classical coordinates, the same lighter tendency appeared at the cervical and middle thirds, with mean L* differences of 1.65 and 1.29, and reversed at the incisal third, where the scanner coordinates were darker by a mean L* of 1.73. No significance test is reported for these coordinate means, for the reason given in Section 2.8.

## 4. Discussion

### 4.1. Principal Findings

This study was designed to describe the distribution of natural tooth colour in a cohort of dental students and, above all, to establish whether a dedicated spectrophotometer and an intraoral scanner can be used interchangeably when the shade of an anterior tooth has to be recorded. Two results stand out. First, the level of agreement between the VITA Easyshade V and the 3Shape TRIOS 5 was fair at best once chance had been taken into account, and on the VITA Classical scale, it deteriorated markedly from the cervical towards the incisal third. Second, and independently of any single agreement coefficient, the scanner showed a consistent tendency to record teeth as lighter than did the spectrophotometer, a directional bias that was statistically unambiguous and clinically meaningful. Translated into colourimetric terms by way of the published tab coordinates, the shades chosen by the two devices differed by a CIEDE2000 colour difference that exceeded the accepted clinical acceptability threshold for the large majority of the assigned tab pairs, and the coordinate comparison indicated that this gap was carried mainly by the lightness axis. Taken together, the two instruments appear to measure the same tooth without returning the same answer, and the discrepancy is systematic rather than random [40,42,45].

### 4.2. Reading Agreement Correctly: Exact Match and Ordinal Proximity

A superficial reading of the Cohen’s kappa for the body shade, which sat close to zero, would suggest that the two devices agreed no better than chance. That interpretation would be wrong, and the reason is instructive. Kappa is known to behave paradoxically when the observations are concentrated in a small number of categories, because the expected agreement it subtracts becomes very large and the coefficient is driven towards zero even when the two raters frequently select the same tab [63]. In our sample the great majority of teeth fell within a narrow, light band of the shade space, which is exactly the situation in which this paradox operates. When agreement was quantified instead with coefficients that are robust to skewed marginal distributions, namely the Gwet’s AC1 and the Brennan–Prediger coefficient, agreement moved into the slight-to-fair range rather than to nil, and these values were consistent with the quadratic-weighted kappa and with the intraclass correlation coefficient [58,59,61,81]. The observed agreement itself tells the same story: a quarter of the teeth matched exactly on the Classical body shade, and roughly half fell within one shade-guide unit, so the devices are neither identical nor unrelated [82,83]. Reporting a family of coefficients rather than a single number is therefore not statistical decoration but a safeguard against a well-documented artefact, and it is the approach recommended for agreement studies of this kind [48,67,84].

### 4.3. Direction and Magnitude of the Inter-Device Bias

Whereas the agreement coefficients are sensitive to how the shades are distributed, the systematic bias is not, and this is what makes it the more robust part of our findings. The Wilcoxon signed-rank test confirmed that the disagreement between the devices had a preferred direction, and the Stuart–Maxwell test that the two marginal distributions differed at every region: the scanner read lighter than the spectrophotometer by a mean of about 1.8 value-ordered units on the Classical scale and by close to one full lightness group on the 3D-Master scale, and it did so in the majority of teeth at every region [64,67,68]. The colourimetric analysis put an approximate perceptual figure on that shift: the mean lightness difference between the tabs chosen by the two devices was roughly four L* units on the 3D-Master coordinates, and the resulting CIEDE2000 colour differences lay above the acceptability threshold for most of the assigned tab pairs. Because those differences are derived from published tab coordinates rather than measured on the teeth, the perceptual relevance of the bias is an estimate, but one that points in the same direction as the purely ordinal analysis, which depends on no coordinate source [78,79,85]. The Bland–Altman plot suggested that the bias was not constant but widened for the darker teeth, and regression of the between-device difference on the mean of the two readings confirmed this, with a slope of −0.580 (95% confidence interval −0.809 to −0.351) at the body region and comparable slopes at the other two regions. Two qualifications are necessary. First, because the scanner readings are compressed against the light end of a bounded ordinal scale, part of this proportional pattern is the expected consequence of regression to the mean, so the magnitude of the effect is probably overstated. Second, the pattern is not uniform along the tooth: at the incisal third, the offset was small and the dispersion the widest of any region, so what the two instruments produce there is scatter rather than a correctable bias. Where the bias is genuinely systematic, at the cervical and middle thirds, the two instruments diverge most precisely where an accurate reading matters most for a natural-looking restoration. A difference of this size and direction cannot be dismissed as measurement noise, and it means that a shade captured on one device should not be transferred to the laboratory as though it had come from the other [41,43].

These findings can be placed against the only quantitative synthesis of this comparison currently available, the 2025 systematic review and meta-analysis of intraoral scanner against spectrophotometer shade matching, which screened 23 eligible articles and pooled 11 of them [40]. That review reported a pooled trueness, the proportion of scanner readings agreeing with the spectrophotometer, of between 0.28 (95% confidence interval 0.09 to 0.60) and 0.38 (0.24 to 0.53), against a pooled repeatability of between 0.81 (0.64 to 0.91) and 0.85 (0.74 to 0.92), and concluded that shade determination with an intraoral scanner is not recommended. The exact agreement observed here is of the same order: 33.1% at the cervical third lies within the pooled range and 25.0% for the Classical body shade a little below it, both within the reported confidence intervals, and the direction of the present finding is the same. A comparison of magnitudes must nevertheless be made with caution. The pooled values are proportions of matching designations, drawn from studies that differ in the scanner used, in the shade system and in whether the comparison was made in vivo or in vitro; the present cohort is younger and lighter than those of most of the pooled studies; and the colour differences reported here are derived from published tab coordinates rather than from directly measured coordinates, so they do not stand on the same footing as the colourimetric values reported by the individual studies in that review. The specific contribution of the present study to that literature is the pairing of the TRIOS 5 with the Easyshade V in vivo, at three regions and in both shade systems simultaneously, a combination that the review identifies as sparsely covered.

### 4.4. Mechanisms: Colour Capture, Tab-Mapping Algorithms and Dehydration

Two features of the scanner data indicate where much of the difference originates. The scanner placed a large share of teeth at the light end of both scales and assigned bleached designations to roughly one participant in six, a category the spectrophotometer almost never used. A young cohort genuinely does have light teeth, so part of this shift is real, yet its magnitude and consistency point to the way the scanner acquires and processes colour rather than to the teeth alone. An intraoral scanner reconstructs colour from camera sensors and on-screen rendering under an internal illumination that is optimised for capturing geometry, and the step that maps the captured colour to a discrete VITA tab is an internal algorithm that can carry a bias of its own, so scanner-derived shades have repeatedly been reported to skew towards lighter and less chromatic values than those of contact spectrophotometry [26,57,86,87,88,89]. Ambient lighting and the spectral character of the illuminant are further recognised sources of variability for instrumental tooth colour, even though the operatory lighting was controlled here [57,87,90].

A second mechanism deserves attention rather than dismissal. Teeth lose water and become lighter and more opaque within minutes of isolation and air exposure, and al-though the order of the two devices was randomised, an intraoral scan takes longer than a single spectrophotometric reading and involves continued air drying of the labial surface, so progressive dehydration during the appointment could have raised the value recorded by the scanner [91]. The direction of this effect is testable in principle, and randomising the order does not by itself remove it. If the scan precedes the spectrophotometric reading, the scan dries the tooth and the spectrophotometer then reads an already-dried surface, so the two readings should converge; if the scan follows, the tooth is drier when it is scanned and the gap should widen. A between-device difference of similar magnitude in both sequences would therefore be strong evidence against the dehydration explanation. The realised randomisation sequence was not recorded in the analysis dataset, so this order-stratified comparison and the device-by-order interaction test could not be carried out here, and the mechanism remains open rather than excluded. The protocol nevertheless limited the opportunity for dehydration to operate, through the minimal isolation, the ten-minute rehydration and the fixed five-minute interval between readings described in Section 2.5 [55]. This mechanism is unlikely to explain the whole effect, because the bias was large, was present in both shade systems and reached bleached designations that brief dehydration alone would rarely produce, but it cannot be excluded and should be controlled in future work by standardising isolation time and allowing rehydration between the two measurements.

### 4.5. Regional Variation and the Challenge of the Incisal Third

Agreement was not uniform across the tooth. On the VITA Classical scale, it was best at the cervical third and effectively collapsed at the incisal third, where both the kappa and the rank correlation approached zero. A near-zero mean signed difference accompanied by the widest spread of any region is clinically distinct from a consistent bias: it cannot be corrected by applying a fixed offset, and it means that an incisal reading taken with either device may fall on either side of the other by a substantial margin. This is also why the incisal third combines the smallest bias with the largest colour difference and with the absence of agreement, three findings that appear contradictory only if bias and scatter are not distinguished. This regional pattern is consistent with the optical behaviour of incisal enamel, which is thin and highly translucent, sits against the dark backdrop of the oral cavity, and presents an apparent colour that depends heavily on how a given instrument handles translucency and edge effects [92,93,94]. The colourimetric results reinforced this reading, because the incisal third produced the largest colour differences of any region and the only reversal of the lightness bias. The two shade systems even ordered the regions differently, the Classical value rank identifying the cervical third as the darkest and the 3D-Master lightness group the incisal third, an internal contradiction of the kind that appears where translucency rather than body colour drives the reading. For the clinician this is a reminder that the incisal region is precisely where instrumental shade matching is least dependable and where visual judgement and a layered restorative approach continue to matter [95,96]. It also cautions against reducing a tooth to a single body shade, since the cervical-to-incisal gradient is a real and measurable feature of natural teeth that has been quantified directly in colourimetric studies of anterior teeth [97,98,99].

### 4.6. Distribution of Natural Tooth Colour

The distribution of shades in this cohort agrees closely with the wider literature on natural tooth colour. Light A-group shades dominated, and the cervical third was consistently more chromatic than the incisal third, a pattern that has repeatedly been reported both in young adults and in larger population samples measured by spectrophotometry in several countries [97,98,100,101,102]. The concentration of the sample within a narrow and light region of the shade space is characteristic of a young population, since teeth tend to darken and become more chromatic with age and differ modestly by sex, and it is precisely this feature that makes the chance-corrected agreement coefficients sensitive to the base-rate paradox, which is why the shade distribution and the agreement analysis have to be interpreted together [63,103,104].

### 4.7. Clinical Implications and the Interchangeability Question

The practical weight of a colour difference in dentistry is judged against perceptibility and acceptability thresholds derived from observer studies, and a difference of one shade-guide unit or one 3D-Master lightness group generally lies beyond the 50:50 acceptability threshold [77,78,105,106,107,108]. The CIEDE2000 difference estimated here exceeded that threshold for most of the assigned tab pairs; because the estimate is derived from shade designations, it describes the colour distance between the tabs the instruments chose rather than a measured difference between the teeth. A colour error of this size at the shade-selection stage has been reported to translate into a visibly mismatched final restoration [109,110], although no restorations were fabricated and no patient or observer assessments were made in the present study. The safe clinical rule that follows is to select one device and use it consistently, from the initial shade selection through to the try-in and final verification, so that any residual instrument bias is at least held constant across the workflow [111,112]. The present data do not identify which of the two instruments lies closer to the true colour of the tooth, because no independent reference standard was measured, and no such claim is made. What the literature does support is that the contact spectrophotometer has the better documented repeatability of the two device classes, which is a property of precision and not of accuracy, while the scanner is best regarded as a convenient means of colour communication whose lighter tendency must be kept in mind [17,21,113].

### 4.8. Strengths and Limitations

This study has real strengths. It used paired clinical measurements from both devices on the same teeth, in both shade systems and at three regions, in a cohort of reasonable size, and it analysed agreement with tools appropriate to categorical and ordinal shade data rather than treating shades as if they were continuous quantities. The combination of exact and weighted agreement, the intraclass correlation, the paradox-robust coefficients, the Stuart–Maxwell test and a Bland–Altman analysis of the value ranks gives a far more complete and honest picture than any single agreement percentage would, and the reporting follows the recommendations for reliability and agreement studies [48,63,84].

The limitations should temper how far the results are carried. Only the maxillary right central incisor was measured, in a single centre, in a young and fairly homogeneous group of dental students, so the distribution findings in particular may not transfer to older or more varied populations [98,103]. Dental students are young, are selected for an interest in oral health and maintain stricter oral-hygiene habits than the general population, so their teeth are lighter and less chromatic than those of an unselected adult sample. The concentration of readings at the light end of both scales, and with it the base-rate behaviour of the chance-corrected coefficients, must therefore be expected to differ in a general clinical population, and neither the shade distribution nor the agreement estimates reported here should be transferred to such a population without replication. The protocol reinforces that restriction. Every tooth was polished before measurement, so surface stain and acquired pellicle had been removed from all of them, and what is reported here is the shade distribution of cleaned enamel rather than of teeth as they present in routine practice. The surface preparation is therefore a third contributor to the light distribution, alongside the age and the selection of the cohort. The most important methodological caveat concerns the colour-difference analysis. The devices recorded discrete tab designations rather than exporting a raw spectral L*a*b* value for each tooth, so the CIELAB and CIEDE2000 differences reported here were obtained by mapping each designation to the published mean coordinates of that tab measured with a reference spectrophotometer [72,73]. The VITA Easyshade V can display CIE L*a*b* coordinates, as stated in Section 2.4, but they were not transcribed, because the case-report form was built around the tab designations for the reasons set out in Section 2.8, so the limitation is one of the study dataset and not of the capability of the instrument. This approach expresses the colour distance implied by the two devices choosing different tabs, which is the quantity that matters for shade communication, but it inherits the sampling and averaging of the coordinate source and does not capture within-tab variation of the individual tooth. The coordinate source, moreover, did not state the illuminant and standard observer used for its measurements, so the absolute colour-difference magnitudes should be read as indicative rather than exact, even though the direction and the region-by-region pattern are unaffected by this [74,80].

Exporting raw per-tooth coordinates from both devices in future work would permit a fully tooth-specific CIELAB and CIEDE2000 analysis and a whiteness-index comparison [75,85,114,115,116,117]. Each tooth in the analysed dataset was measured once, so intra-device repeatability, which is itself part of what makes a device trustworthy, was not quantified, even though repeatability of both spectrophotometers and scanners has been examined elsewhere [36,113,118,119,120,121,122]. This bears on how the agreement coefficients should be read. Part of the disagreement between the instruments must reflect the within-device variability of each of them, and without repeatability estimates, the two components cannot be separated. The conclusion that the devices are not interchangeable is not weakened by this, since an instrument that is unreliable in itself is no more interchangeable than one that is reliably biased; what cannot be resolved from the present data is the magnitude of the true between-device bias, and the coefficients reported here, which absorb that within-device noise, should be regarded as lower bounds on what perfectly repeatable instruments would give. The realised order of the two instruments was not recorded in the analysis dataset, a gap whose consequence is discussed in Section 4.4. A single trained examiner took and recorded all readings, so inter-examiner variation and formal blinding could not be evaluated [18,54]. The two devices are not symmetrical in this respect. On the spectrophotometer, the measured region is fixed physically, by where the probe tip is placed against the tooth, whereas on the scanner, the three zones are selected by the examiner on the reconstructed labial surface after acquisition. That selection is a subjective step which has no counterpart on the spectrophotometer, and its influence should be greatest at the incisal third, where the boundary of the zone is hardest to place against a translucent and progressively thinning enamel edge. It is a plausible contributor to the dispersion observed at that region and it cannot be separated from the instrumental sources with the present data. The calibration exercise described in Section 2.6 was procedural and produced no coefficient, so intra-examiner reproducibility was not quantified either. A further limitation of design, rather than of analysis, is that no human observer arm was included. Visual shade matching with a physical guide by experienced clinicians remains the everyday clinical baseline against which any instrument is ultimately judged, and its absence means that this study can describe how the two instruments relate to each other but not how either relates to the judgement a clinician would make at the chairside; the clinical extrapolation of the findings is limited accordingly. Finally, the handling of the scanner bleached designations on an extended light end of the value scale, although reasonable, is not the only defensible choice; the sensitivity analysis reported in Section 3.3 shows that this convention accounts for at most about a quarter of the Classical bias and changes neither its direction nor its significance.

### 4.9. Future Directions

The most obvious direction is to export raw per-tooth L*a*b* coordinates from both devices so that colour differences can be computed for each individual tooth with the CIEDE2000 formula rather than through published tab coordinates, which would refine the colour-difference estimates reported here and remove their dependence on an external coordinate source [75,77,80]. Repeated measurements would allow the intra-device repeatability and the inter-examiner reliability of both instruments to be quantified alongside their agreement [36,113]. A three-arm design that adds a panel of experienced clinicians matching each tooth visually against a physical VITA guide under standardised lighting would place both instruments against the clinical baseline they are intended to replace, and would show whether the lighter scanner readings are also detectable by the human eye [9,10,16]. Recording the realised order of the two instruments, together with the elapsed time from isolation to each reading, would permit the order-stratified analysis and the device-by-order interaction test that the present dataset cannot support, and would settle the contribution of dehydration to the observed bias. Extending the design to several teeth, to older and more diverse patients, and to restored as well as natural teeth would test the generalisability of both the distribution and the bias, and following the recorded shades through to the appearance of the finished restoration would connect these instrumental differences to the outcome that ultimately matters to the patient [95,111].

## 5. Conclusions

Within the limits of this cross-sectional study, the VITA Easyshade V spectrophotometer and the 3Shape TRIOS 5 intraoral scanner produced shade readings that agreed poorly once chance was accounted for, and the scanner read teeth as systematically lighter than the spectrophotometer, most clearly at the cervical third, while at the incisal third, the systematic offset was small and the dispersion of the differences was the widest of any region. The two devices should not be regarded as interchangeable. When colour has to be documented and reproduced within a single case, one instrument should be chosen and used throughout. The study measured agreement and relative bias and not accuracy: no independent reference standard was included, so it cannot be concluded that either device lies closer to the true colour of the tooth. The colour differences reported are estimates of the distance between the assigned tabs rather than direct colourimetric measurements of the teeth, and the expectation that a discrepancy of this magnitude would be visible in a finished restoration, though supported by the previous literature, was not tested here. The intraoral scanner remains convenient as part of a digital workflow, and its shade function is useful for colour communication, provided its lighter bias is kept in mind.

## Figures and Tables

**Table 1:** Exclusions from the shade-designation-derived colour-difference computation, by shade system, region and reason. Scanner bleached designations (0M1 to 0M3) have no entry in the VITA Classical coordinate table and could not be mapped; a small number of designations fell outside the published coordinate tables. Because a colour difference requires a mappable designation from both instruments, a tooth is excluded when either reading cannot be mapped. All exclusions affect the colour-difference computation only, the agreement analysis of Table 3 being based on all available paired readings.

Shade System and Region	Teeth Measured	Teeth Analysed	Excluded (n)	Scanner Bleached Designation, no VITA Classical Coordinate (n)	Designation Outside the Published Coordinate Tables (n)
**VITA Classical**					
Cervical	146	141	5	4	1
Middle third (=body shade)	146	119	27	25	2
Incisal	146	134	12	11	1
**VITA 3D-Master**					
Cervical	146	143	3	0	3
Middle third (=body shade)	146	145	1	0	1
Incisal	146	146	0	0	0

**Table 2:** Characteristics of the study population (n = 146 teeth; age, sex and residence available for 142 participants).

Characteristic	Value
Teeth measured (participants)	146
Age, years, mean (SD) [range]	20.3 (1.3) [18 to 23]
Sex, women/men, n (%)	89 (62.7)/53 (37.3)
Cohort, Romanian/international, n (%)	113 (77.4)/33 (22.6)
Residence, urban/rural, n (%)	120 (84.5)/22 (15.5)

## Data Availability

The de-identified data supporting the findings of this study, and the Python code used to compute the Gwet’s AC1 and Brennan–Prediger coefficients, are available from the corresponding author on reasonable request.

## Supplementary Materials

The following supporting information can be downloaded at: https://www.mdpi.com/article/10.3390/dj14100636/s1, Table S1: Colour differences implied by the shade designations of the VITA Easyshade V and the 3Shape TRIOS 5, by region and shade system; Figure S1: Mean CIEDE2000 colour difference implied by the shade designations of the two devices by tooth region and shade system.
