# Supplementary material for: Digital Tooth Colour Acquisition: Agreement Between Spectrophotometric and Intraoral Scanner Measurements in Dental Students
Source: Dent J (Basel). 2026 Oct 4;14(10):636. doi: 10.3390/dj14100636 (PMC13703656; doi:10.3390/dj14100636)
Supplement: Supplementary file 1 [file dentistry-14-00636-s001.zip › dentistry-4527640-supplementary.pdf]

Supplementary Materials

Table S1 and Figure S1 report the colour differences implied by the shade designations of the two devices in full, for readers who wish to consult them. They are to be published as Supplementary Material.

Table S1. Colour differences implied by the shade designations of the VITA Easyshade V and the 3Shape TRIOS 5, obtained by mapping each recorded designation to published CIE L*a*b* tab coordinates, by region and shade system. Values are the mean CIEDE2000 (ΔE00) and CIELAB (ΔE*ab) colour differences with standard deviations, the percentage of assigned tab pairs whose published coordinates differ by more than the 1.8-unit CIEDE2000 acceptability threshold, which is not the same quantity as a percentage of teeth judged unacceptable, and the mean coordinate differences (TRIOS minus Easyshade) for L*, a* and b*; a positive ΔL* indicates lighter scanner readings. The standard deviations describe the variability of the assigned tab pairs and not the variability of tooth colour, and the numbers analysed differ between the shade systems for the reasons set out in Table 3. Three points should be carried with this table when it is read on its own. First, the VITA Classical rows are computed after excluding the teeth for which the scanner returned a bleached designation, which the Classical coordinate table cannot represent; those are the teeth on which the two instruments diverged most, so the Classical values understate the difference between the devices, most markedly at the middle third, and they should not be read as evidence of closer agreement in that system. Second, the sign convention is the reverse of that in Table 2 of the main text: there a negative mean difference indicates a lighter scanner reading, whereas here a positive ΔL* indicates the same thing, because Table 2 is computed on a value-ordered rank that increases towards the darker tabs while L* increases towards the lighter. Third, only the CIEDE2000 differences are benchmarked against a threshold here, although Section 2.8 of the main text gives dentistry-specific thresholds for both metrics. CIEDE2000 is the metric on which the interpretation in this paper rests, and the CIELAB differences are reported alongside it for comparability with the older literature rather than as a second, separate test against a threshold; the corresponding CIELAB percentages were accordingly not computed.

Region	n	ΔE00 (SD)	ΔE*ab (SD)	> AT (% of pairs)	ΔL*	Δa*	Δb*
VITA 3D-Master							
Cervical	143	3.83 (2.26)	5.59 (3.15)	82	+3.65	-0.76	-2.34
Middle	145	4.15 (2.20)	5.93 (3.17)	88	+4.37	-0.50	-2.07
Incisal	146	4.82 (3.18)	6.55 (4.19)	86	+3.86	-0.60	-2.92
VITA Classical							
Cervical	141	2.32 (2.17)	3.40 (3.18)	61	+1.65	-0.38	-1.52
Middle	119	2.15 (1.86)	3.21 (2.79)	57	+1.29	-0.43	-1.98
Incisal	134	3.52 (2.27)	4.81 (3.09)	66	-1.73	-0.18	-1.73

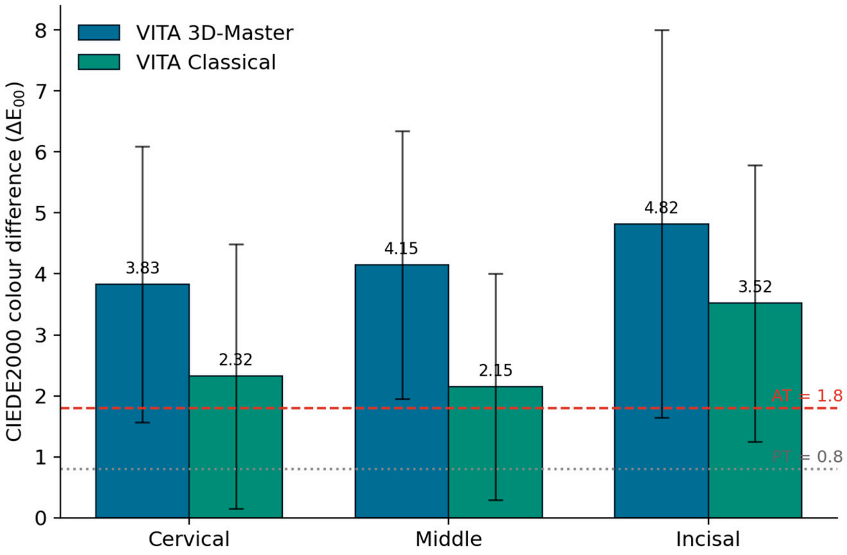

Figure S1. Mean CIEDE2000 colour difference implied by the shade designations of the two devices by tooth region and shade system. Error bars are one standard deviation; as in Table S1, those standard deviations describe the variability of the assigned tab pairs and not the variability of tooth colour. The dotted line is the perceptibility threshold (PT = 0.8) and the dashed line is the acceptability threshold (AT = 1.8) for dentistry; because the values are derived from published tab coordinates, the comparison with these thresholds is an estimate.
